# Healthcare Providers and Caregivers’ Perspectives on the Quality of Child Health Services in Urban Indonesia: A Mixed-Methods Study

**DOI:** 10.3390/ijerph18158047

**Published:** 2021-07-29

**Authors:** Agus Setiawan, Poppy Fitriyani, Rizkiyani Istifada, Shefaly Shorey

**Affiliations:** 1 Department of Community Health Nursing, Faculty of Nursing, Universitas Indonesia, Depok 16424, Indonesia; poppy@ui.ac.id (P.F.); rizkiyaniistifada@gmail.com (R.I.); 2 Alice Lee Centre for Nursing Studies, Yong Loo Lin School of Medicine, National University of Singapore, Singapore 117597, Singapore; nurssh@nus.edu.sg

**Keywords:** children under 5 years old, service, mixed method, healthcare providers, caregivers, urban

## Abstract

Although Indonesia’s child mortality rate has decreased in tandem with the global trend, healthcare services can be further improved for children. This study aims to explore the perceptions of healthcare providers and caregivers of children aged below five years to improve child health in urban Indonesia. A mixed-methods study design was adopted. Quantitative data were collected via questionnaires from the caregivers. Qualitative data were collected via semi-structured interviews from the healthcare providers. Analysis of 540 caregiver questionnaires regarding the care provided to their child revealed that the majority of them were very satisfied (46.1%) and satisfied (52.2%) with the care received. Analysis of 12 interviews with healthcare providers derived three major themes: (1) importance of one’s role as a healthcare provider; (2) factors affecting the delivery of healthcare; and (3) the way forward in caring for young children. Although caregivers were very satisfied with the care received for their children, the perception of healthcare providers regarding their responsibility in delivering care to children under five years old provides insights into improving the quality of services for children in Indonesia. Our findings suggest that mortality and morbidity of children aged below five years can be best reduced by optimizing primary and secondary preventive measures.

## 1. Background

Childhood mortality rates have decreased worldwide by almost 60 percent in the last three decades [1,2]. Sub-Saharan Africa and South Asia are among the regions that mostly contribute to the number of child deaths [2,3]. The majority of deaths of children aged below five years were predominantly from preventable causes [3]. In a recent report by the World Health Organization, children from poorer households in urban areas from underdeveloped and developing countries had a significantly higher mortality rate than children from richer households [4]. Children from poor households in urban areas had limited healthcare accessibilities similar to children in rural areas [4]. Therefore, more accessible, affordable, and quality healthcare could contribute to further reducing child mortality.

The 2017 demographic and health survey in Indonesia reported that in the last five years, the number of reported deaths for children under the age of five was 32 deaths per 1000 live births, slightly decreased from 36 deaths per 1000 live births [5]. The decrease in childhood mortality could be due to several factors, such as improvement in access to healthcare, education level of parents, and socio-economic status. However, a recent review has highlighted that Indonesian children’s health is under risk due to contaminated water, air, and food as well as health care disparities [6]. Therefore, to meet child survival rate as targeted in Sustainable Development Goals (SDGs), the efforts must be tailored toward not only sustaining current trends but also accelerating the progress. This can possibly be done by strengthening the health system through improving the quality of care as well as engaging caregivers so that high-impact preventive and curative intervention can be appropriately provided [3]. 

Previous research reported that mortality and childbirth rates are influenced by healthcare services [7]. Considering the influence of healthcare services on childhood mortality, the Indonesian government is committed to increasing the number of primary health centers and revitalizing community health centers to improve access to healthcare services for families with young children [8]. Healthcare services at the local community health centers mainly determine the quality of healthcare services for children under five years old and are carried out in accordance with the Healthy Indonesia Program (*Program Indonesia Sehat*) [9]. However, the government’s effort to increase the number of primary health centers may not equate with the increase in the quality of services provided and thus needs to be evaluated. A recent Indonesian health and demographic survey revealed that the under-five childhood mortality rate in communities with a low socio-economic status may increase to as high as 52 deaths per 1000 live births [5]. In this regard, the quality of healthcare services available for families with young children in Indonesia must be assessed [10].

Primary healthcare in Indonesia has been revitalized to improve public access to services. Healthcare services to children aged below five years at the community health centers include integrated services for children under five (*posyandu balita*), immunization services, and integrated management for sick children (*Manajemen Terpadu Balita Sakit*) [11]. According to the Alma-Ata Declaration, primary healthcare consists of eight essential components, one of which is providing healthcare for mothers and their children [12]. Under the Helsinki Statement, pursuing health equity (which includes striving for equitable access to healthcare) is a fundamental human right that should be fulfilled [13]. Improving the availability of primary healthcare can improve the effectiveness of health services, the healthcare budget, and the gaps in healthcare that still persist today [14]. This is supported by previous studies, wherein better healthcare at the primary level will ultimately lead to a healthier community investment and a sound budget [15,16,17]. Programs in healthcare are highly dependent on the role of healthcare providers in carrying out their duties. Therefore, the number of qualified healthcare providers should be proportional to the overall population. The 2019 Indonesian Health Profile Report showed that 136,203 healthcare providers consisting of 17,954 doctors and 118,249 nurses and midwives were spread over the community health centers throughout the nation [18]. The figure showed that, based on quantity alone, the proportion should be further improved, i.e., the number of healthcare providers in community health centers remains inadequate. Moreover, the efforts to revitalize community health centers has not met the projected increase in quality of primary health service, especially when the role of healthcare providers has been found to be an important factor that achieves quality healthcare services to children aged below five years. Consequently, the perception of healthcare providers with regard to healthcare services available for children under the age of five years and how they feel being the frontline care providers are crucial to improve services at the community health centers by filling the gaps highlighted by the providers.

In addition, parents’ help-seeking behavior plays a pivotal role in utilizing health services. A study conducted in rural Indonesia shows that one of the challenges is that parents do not wish to receive health-related interventions due to discrepancy in their beliefs and support provided by the health providers. In contrast, parental access to healthcare have been found to improve with an increase in their knowledge and satisfaction with the care received [19]. This highlights the importance of positive caregiver experiences with the healthcare services to influence the service usage. However, little is known about the experience of caregivers and healthcare providers in receiving healthcare, especially in urban Indonesia. Therefore, this study aims to fill this gap by exploring the perceptions of both caregivers and healthcare providers regarding healthcare services provided to children under the age of five in urban Indonesia. It is expected that this study’s findings will enable related stakeholders to improve healthcare services for children based on caregivers’ and healthcare providers’ needs. 

## 2. Method

### 2.1. Research Design

This study employed a mixed-methods approach. Quantitative data on the experiences of caregivers and satisfaction of parents with healthcare provided to their children were collected using questionnaires developed by the authors. Qualitative data were collected from healthcare providers via in-depth, semi-structured interviews. An interview guide was used to collect data, which were developed based on literature and authors’ clinical expertise. Data collection was conducted from August 2019 to September 2019.

### 2.2. Population and Study Setting

The study was carried out in six community health centers in Depok, an urban city in Indonesia where the University of Indonesia is located. The inclusion criteria were healthcare providers who have (1) worked in a primary health center for at least 6 months and (2) have cared for children aged below 5 years. The participants for the qualitative component of the study were selected using a purposive sampling technique so that doctors and nurses/midwives could be included in the interviews [17]. The inclusion criteria for caregivers were primary caregivers who lived with the child and accompanied the child to community health centers. The convenience sampling technique was used to recruit the caregiver in this study.

### 2.3. Data Collection

Healthcare providers and caregivers who met the inclusion criteria were approached by the researcher at the healthcare centers. The study details were explained thoroughly, and those who showed interest provided written informed consents for participation. For caregivers, a quiet room was allocated to fill up the questionnaires. The Service Provision Assessment Questionnaire (SPAQ) from the Demographic and Health Surveys Program [20] was adopted by the authors and included questions such as: “How satisfied are you with the care your child receives from the community health center?”. The questionnaires can be found in the online Supplementary material (Table S1). The interviews with caregivers were conducted after their children received the care and before they left the health centers. The average time taken to complete the questionnaire was less than 10 min. For the healthcare providers, a mutually agreed date and place of interview was prearranged, and face-to-face individual interviews were conducted. Demographic data from healthcare providers and caregivers were also collected. All data were collected in Bahasa Indonesia and were translated to English by bilingual authors.

### 2.4. Data Analysis

Descriptive quantitative method using SPSS version 27 (IBM Corp., Armonk, USA) was used to analyze caregivers’ data, and the results were presented using frequencies and percentages. Thematic analysis was used to evaluate qualitative data. Data were recorded, transcribed, and analyzed following the stages of Braun and Clark’s (2006) thematic analyses [21]: (i) the researcher reviewed the interviews three to five times; (ii) after reviewing the transcripts several times, the researcher searched and coded the key and important points from the interviews; (iii) the researcher grouped these codes into sub-themes; (iv) the researcher consolidated sub-themes describing similar phenomenon into themes; and (v) the researcher validated the result of the research description with the participants [22,23]. Two researchers independently reviewed the transcripts and conducted the thematic analysis. Frequent meetings were held to gain consensus. Any discrepancies were resolved by consulting with a third researcher. The rigor of the data was maintained via credibility, dependability, and confirmability. Credibility was ensured by deep immersion into data, that is, the researchers read the participants’ transcripts several times to ensure that the emerging themes were a true representation of the participants. Dependability and conformability were maintained by keeping the audit trail such that all the decisions made by the researchers from data collection to analysis were documented. 

### 2.5. Research Ethics

All subjects gave their informed consent for inclusion before they participated in the study. The study was conducted in accordance with the Declaration of Helsinki, and the protocol was approved by the Ethical Committee of the Faculty of Nursing, Universitas Indonesia (Reference number: SK-185/UN2.F12.D1.2.1/ETIK.FIK.2019). 

## 3. Results

Five hundred and forty caregivers from six community centers participated in the study. The demographic data and roles of the caregivers are presented in Table 1 and Table 2, respectively. The majority of the caregivers were very satisfied (46.1%) and satisfied (52.2%) with the care provided to the child under their care. They had no issues with waiting time (54.6%), availability of medicines (93.5%), cleanliness of the facility (90.4%), and fee charged for the care received (98.7%). They were also satisfied (91.1%) with the way the staff cared for their child but hoped to receive (57.6%) more detailed information regarding their child’s condition. Almost half of the parents (53.5%) wished to have more privacy when assessments were carried on their children. 

For qualitative interviews, 12 participants consisting of six doctors, five nurses, and one midwife participated. The interviews lasted from 30 min to 45 min each. Table 3 presents the characteristics of healthcare providers who participated in the study. 

The three themes that emerged from the data analysis were (1) importance of one’s role as a healthcare provider, (2) factors affecting the delivery of healthcare, and (3) the way forward in caring for young children.

### 3.1. Theme 1: Importance of One’s Role as a Healthcare Provider

The majority of healthcare providers found their role to be very important in delivering quality care to children under the age of five years. They find meaning in delivering healthcare, which they see as part of their responsibilities and efforts to prevent the death of children under their care. Most of the healthcare providers believed that caring for young children and preventing them from dying is part of their professional responsibility.

“My main job as a nurse here in the clinic is to provide care to sick children, promote their wellbeing. I hope we can contribute to reduce the (child) mortality in the city”.

(Participant 6, a nurse)

The healthcare providers also strongly acknowledged that a patient has the right to receive the best care possible.

“Provide the best service…until the family understands what they should or should not do to the patient (children under five)”.

(Participant 2, a doctor)

Healthcare providers assigned to a community health center were aware that healthcare services to children under five years old at the center are part of preventive measures to minimize the occurrence of severe illnesses and reduce child mortality in a specific area.

“We are making a judgment call, we can assess whether something is wrong with a child or not”.

(Participant 1, a nurse)

The healthcare providers proudly shared the efforts they put in to improve the quality of healthcare for the children, which included providing education, coordinating, and actively participating in training programs. As they felt the importance of their job, they put great effort into supporting the care of the young children, as shared by one of the participant as follows:

“We do not limit our care on location; for the public, we even go to *posyandu* (integrated health services) for weighing and immunization, which may be done outside the building every month”.

(Participant 3, a midwife)

### 3.2. Theme 2: Factors Affecting the Delivery of Healthcare 

The healthcare providers shared various factors that influenced their delivery of care to the children under the age of five years. These factors included the following: (i) the need for following the standard operating procedures (SOP); (ii) internal motivators, such as providers’ confidence and view about their competence and commitment to provide the best quality care to the children; (iii) and having necessary resources, such as educational pamphlets, and a good flow of services (from registration to the delivery of prescriptions).

The healthcare providers strongly believed that SOPs influenced the quality of care that the children received. They felt that if the SOPs can ensure that waiting time can be reduced, then it will enhance the quality of care and services to the young children.

“Quality service…waiting hours, these are two major things to be addressed. If there is a SOP, that would be great”.

(Participant 2, a doctor)

According to the healthcare providers, they should possess the necessary competency to ensure they provide safe and quality care. The healthcare providers also felt that providing best quality care for the children is a form of obligation in carrying out their duties. 

The participants said:

“We must have good knowledge about children’s health care. It is holistic in nature”.

(Participant 8, a doctor)

“I strongly believe that competent health providers are a must to ensure quality care for our patients”.

(Participant 7, a nurse)

“We must stay in course, we must remain up-to-date…we must put the interest of the child first”.

(Participant 9, a nurse)

The majority of the healthcare providers believed that staying committed is one essential aspect of improving the quality of healthcare for children under five years old.

“If you could manage it well (i.e., delivering good care), actually if you were committed, then everything would be all right”.

(Participant 8, a doctor)

Some healthcare providers also felt that having a committed disposition toward one’s work also opened opportunities to collaborate with other healthcare providers. They felt that initiating collaborations would provide the best care for the children.

“If everyone is committed, then everybody would do their work correctly. Therefore, this will ensure that there are less issues during collaboration to provide best care to the children”.

(Participant 9, a nurse)

Majority of the healthcare providers highlighted that, in addition to personal factors (e.g., internal motivators), necessary resources, such as informational materials and supportive facilities with good workflow, were essential to enhance the quality of care for the children.

“We should be provided clear information so that we can guide the parents accordingly…for example, we can provide pamphlets on the importance of seeing doctor every two weeks… timely appointments are very important…”.

(Participant 10, a doctor)

### 3.3. Theme 3: A Way Forward in Caring for Young Children

The healthcare providers shared a wish list that they felt could enhance the quality of care for children in Indonesia. They hoped for better resources and facilities, such as having a playground, a separate treatment facility, and more informed parents. 

One of the nurses highlighted that having a playground in community healthcare centers will reduce fear among children:

“Build a children’s playroom, with books and toys for them to reduce fear of healthcare facilities”.

(Participant 9, a nurse)

All the healthcare providers highlighted the need of separating the examination facilities for children under five years old to prevent contamination or transmission of viruses and bacteria from adult patients while waiting to be examined.

“The children should have a separate examination room equipped with facilities to measure their height and weight, everything should be there… so that we don’t run around and mix patients… potential for infection…”.

(Participant 12, a nurse)

A nursing room is one of the important facilities suggested to improve the quality of healthcare for children under five years old.

“There should be a nursing room located close to the examination room”.

(Participant 10, a doctor)

One of the healthcare providers realized the importance of feedback from the patients regarding their satisfaction. Therefore, healthcare facilities should provide suggestion boxes so patients could provide their feedback regarding the services they received.

“There should be a suggestion box... so that we can learn and improve”.

(Participant 9, a nurse)

Healthcare providers admitted that educational efforts can be enhanced beyond traditional methods, such as handing out pamphlets. Some providers who have begun to use technology, such as social media, to disseminate health information to the public have advocated for its effectiveness

“We provide information (health information) …through the media (technology)…we use Facebook. But more needs to be done”.

(Participant 6, a nurse)

Some of the healthcare providers suggested the need for better coordination between the relevant parties who are involved in the efforts to improve the healthcare for children aged below five years. They suggested to have better coordination among various stakeholders, including community figures, cadres, and local hospitals.

“We are collaborating with community figures, involving community figures, especially the mothers, they, in a sense, are cadres… there should be a middle agency helping in this coordination between parents and us (healthcare providers)”.

(Participant 6, a nurse)

The majority of the healthcare providers felt that coordination across different disciplines were necessary to ensure safety in case of emergencies.

“We have an area of responsibility, especially midwives, so when a medical emergency occurs, they will refer to the appropriate department”.

(Participant 5, a doctor)

Another healthcare provider shared the following:

“When a child is suffering from malnutrition, we will refer that child to a nutritionist”.

(Participant 12, a nurse)

Some healthcare providers reported that good coordination should not be limited to inter-profession or across programs but should also include coordination with leaders or heads of departments in charge of delivering healthcare for children aged below five years.

“There should be a coaching session with the department’s head so that everyone in the department are on the same page”.

(Participant 8, a doctor)

Lastly, all the healthcare providers felt that they should be allowed to attend relevant training to improve the quality of care they can provide for the children.

“A medical worker should keep his/her knowledge and skills up to date”.

(Participant 11, a doctor)

## 4. Discussion

This study aimed to explore the perception of healthcare providers and caregivers regarding the services provided to children aged below five years in urban Indonesia. This study adopted a mixed-methods approach to acquire a comprehensive perception of pediatric healthcare in Indonesia. The majority of the caregivers interviewed in this study were Muslim mothers, which is representative of the country’s demographic profile. The role of caregiving, especially for sick children, is predominantly played by the mothers in Indonesia, and the fathers are considered the sole breadwinners whose main role is to work to fulfill the family needs. Indonesia is the biggest Muslim country in the world, and Muslims account for 87.2% of the country’s overall population [18]. The health providers who participated in the study were mainly young females, which again corresponds to the healthcare providers’ profiles in Indonesia’s primary health system. The young female nurses and midwives are the main health workers who serve the community health centers [18]. Senior health workers are mostly found working in either secondary health services or in the district health office undertaking administrative jobs. 

The majority of caregivers of children under the age of five years were generally satisfied with the healthcare service in Indonesia. Healthcare providers, however, had several suggestions to improve and enhance the healthcare system and delivery of care. Healthcare providers perceived their services to children under five years old as a form of higher calling on top of their regular duty. These findings correspond to the previous local study [19], where committed healthcare providers were appreciated, and parents felt satisfied to receive care from providers who matched their cultural beliefs. This is an important finding highlighting the need of providing culturally sensitive care. The provision of culturally sensitive care has been similarly highlighted towards Black, Asian, and Minority Ethnic (BAME) population in previous research [24,25].

According to the results collected via the questionnaire given to the caregivers, only a select few reported waiting time as a problem. However, many doctors viewed that waiting time should be reduced and that it is a component that adds to the quality of healthcare service. Previous research showed that long waiting times elicit dissatisfaction in patients [26] and that, depending on the patient, their condition could worsen during the waiting time span [27]. These factors make waiting time a key indicator in the quality of healthcare [27]. The reasons for Indonesian caregivers not seeing waiting time as a problem could be that in many developing countries, healthcare providers receive special respect from the public, and as long as they receive some care, the negative experiences, such as waiting time, are often not reported [28]. However, considering the provision of quality healthcare as a basic right of each individual, continuous actions should be implemented to reduce waiting time as a measure to improve the quality of health services.

The majority of the caregivers of children expressed that the explanation they were given by the healthcare providers could be improved. The healthcare providers also noted that educating and providing the patients and their caregivers with adequate information is important in improving the quality of healthcare. A previous study stated that interaction with healthcare providers is usually only 15 min, which is shorter compared with the time spent by a patient in registration, waiting, and prescription collection post doctors’ appointment in clinics [29]. This short time may be insufficient for doctors and nurses to fully explain to the caregivers how to properly monitor the child and answer their questions A study showed that a greater amount of time spent by healthcare providers treating and attending to patients was associated with greater patient and healthcare provider satisfaction [30]. This finding supports this study’s findings, which suggest that healthcare personnel may need to increase the amount of time spent in explaining to the child’s caregiver to increase healthcare service satisfaction. Other than spending more time, which maybe logistically difficult to achieve, the healthcare providers could consider incorporating technology-based, visual/graphic educational materials to inform caregivers on common healthcare-related topics for children aged five and below. Use of adjunct educational materials has been found to be useful among parents of young children [31]; however, this needs to be further evaluated among Indonesian caregivers in future studies. 

Another possible reason why caregivers were not satisfied with the amount of information they received could be due to the lack of caregiver educational qualification on healthcare for children aged under five years. The majority of the caregivers had senior high school or below as the highest educational level, which may not be adequate to understand general healthcare. Therefore, as stated by the healthcare providers, healthcare and medical information should be shared with the general public in more coherent ways. For example, distributing pamphlets are common practice; the public should be further educated by using other more effective educational interventions, such as social media or technology-based educational programs [31,32]. However, the cost-effectiveness and ability to provide up-to-date information of these interventions to caregivers still needs further evaluation among Indonesia parents. Other studies have also found that increased education of caregivers and mothers resulted in improved infant health, survival, and development [33]. Thus, ensuring that the public and the caregivers of children under five years are well educated and informed on healthcare is important. 

The caregivers and healthcare providers wanted improvements in facilities available for children who are receiving healthcare services. More than half of the caregivers stated that privacy was not well maintained for the children, and healthcare providers and suggested separate rooms, waiting areas, and play areas for children to improve healthcare service. This finding is in line with a prior research, which reported that parents expressed dissatisfaction with lack of private facilities to be with their children [34]. Therefore, this suggestion made by the healthcare providers and caregivers could aid in improving healthcare services.

The healthcare providers suggested improving training and coordination among healthcare providers, various healthcare sectors, and cadres to provide improve healthcare service to children aged under five years. A study on increased coordination in primary healthcare found that regardless of the type of strategy used, improving the coordination of care benefits patient outcomes [35]. To ensure safe and evidence-based care delivered to the parents and children, well-informed and trained healthcare providers are strongly encouraged in previous research [36]. Therefore, the inputs from healthcare providers should be considered seriously, and strategies should be developed and implemented to increase the training and coordination of healthcare providers and provide better healthcare services and care for the children.

### Limitations and Implications

This study was limited because only questionnaires were used to collect quantitative data from the caregivers, and qualitative interviews were used to collect data from the healthcare providers due to logistics reasons. Future research should consider collecting qualitative and quantitative data from caregivers and healthcare providers to provide more room for both parties to express specific concerns and suggestions, which are valuable input for improving healthcare services.

Based on the findings, time-management strategies should be improved in healthcare centers to reduce waiting time and ensure sufficient time for medical consultations. Technology-based educational programs could be developed and evaluated for caregivers, and more privacy is needed for caregivers when they visit healthcare centers with the children they care for. Local community centers could organize face-to-face educational workshops weekly or monthly, and nurses or doctors could conduct them. These workshops should have a question-and-answer segment where caregivers of the children can clarify their queries. Educational workshops should also be made readily and frequently available to healthcare providers to update their skills in healthcare-service deliverance and to keep them constantly informed of new healthcare and medical improvements. Inter-disciplinary healthcare coordination could be facilitated using a top-down approach so that necessary and timely arrangements can be implemented. 

## 5. Conclusions

This study reported the perception of caregivers and healthcare providers regarding the status of child health services available for children under five years in urban Indonesia. The findings are important contributions for increasing the quality of the services available for children in Indonesia. Responses from the children’s caregivers and healthcare providers identified areas that need to be improved. Policy makers, healthcare providers, and caregivers should work together to improve the quality of healthcare for children aged below five years and reduce the child mortality rates in Indonesia.

## Figures and Tables

**Table 1:** Demographic data of caregivers.

Characteristics	Frequency	Percentage
Religion		
Muslim	515	95.4
Christian	23	4.3
Catholic	1	2
Buddhist	1	2
Job		
Housewife	463	85.7
Public servant	3	0.6
Private employee	28	5.2
Entrepreneur	28	5.2
Others	18	3.3
Income		
<Regional minimum wage	327	60.6
≥Regional minimum wage	213	39.4
Highest level of Education		
Elementary	50	9.3
Junior high school	121	22.4
Senior high school	286	53
Academy/Diploma	51	9.4
Bachelor	30	5.6
Others	2	4

**Table 2:** Role of caregivers.

Role	Frequency	Percentage
Mother	485	89.9
Father	23	4.3
Uncle/Aunt	10	1.9
Grandparent	20	3.7
Other	2	0.4
Total	540	100

**Table 3:** Characteristics of healthcare providers.

Characteristics	*n* (%)
	Healthcare Providers
Gender	
Male	0 (0)
Female	12 (100)
Age range	26–54 years
Years of experience	
0–5 years	9 (75)
6–10 years	2 (16.7)
>10 years	1 (8.3)
Profession	
Doctor	6 (50)
Nurse	5 (41.7)
Midwife	1 (8.3)
Attended training(s) on child health in the last year	
Yes	10 (83)
No	2 (17)

## Data Availability

The data presented in this study are available on request from the corresponding author. The data are not publicly available due to ethical reason.

## Supplementary Materials

The following are available online at https://www.mdpi.com/article/10.3390/ijerph18158047/s1. Table S1. the DHS Service Provision Assessment Questionnaire (SPAQ).
