# Supplementary material for: Healthcare Providers and Caregivers’ Perspectives on the Quality of Child Health Services in Urban Indonesia: A Mixed-Methods Study
Source: Int J Environ Res Public Health. 2021 Jul 29;18(15):8047. doi: 10.3390/ijerph18158047 (PMC8345802; doi:10.3390/ijerph18158047)
Supplement: Supplementary file 1 [file ijerph-18-08047-s001.zip › ijerph-1312656-supplementary.pdf]

MEASURE *DHS* SERVICE PROVISION ASSESSMENT SURVEY

SICK CHILD CARETAKER EXIT INTERVIEW

FACILITY IDENTIFICATION

Name of the facility: ______________________________

Location of the facility: ______________________________

FACILITY NUMBER . . . . [ ][ ][ ][ ][ ]

PROVIDER CODE. . . . . [ ][ ]

INFORMATION ABOUT INTERVIEW

DATE: ______________________	DAY . . . . [ ][ ]<br>MONTH . . . . [ ][ ]<br>YEAR . . . . [2][0][1][ ]
Name of the interviewer: ______________________	INTERVIEWER CODE . . . . [ ][ ]<br>CLIENT CODE. [ ][ ][ ]

1. Information About Visit - CARETAKER OF SICK CHILD

NO.	QUESTIONS	CODING CLASSIFICATION	GO TO
	**READ TO CLIENT:** Hello, I am_____________. As my colleague mentioned, we are representing [IMPLEMENTING ORGANIZATION]. We are conducting a study of health facilities in [COUNTRY] in order to improve the services this facility offers and would like to ask you some questions about your experiences here today.<br>Please know that whether you decide to allow this interview or not is completely voluntary and will not affect services you receive during any future visit. You may refuse to answer any question, and you may stop the interview at any time.<br>Information from this interview may be provided to researchers for analyses, but neither your name nor the date of services will be on any shared information, so your identity will remain completely confidential.<br>Do you have any questions for me? Do I have your permission to continue with the interview?<br>Interviewer's signature<br>(Indicates respondent's willingness to participate)	DAY [ ][ ] MONTH [ ][ ] YEAR 2 0 1 [ ]	
100	May I begin the interview?	CLIENT AGREES . . . . 1<br>CLIENT REFUSES 2	 → END
101	RECORD THE TIME THE INTERVIEW STARTED	[ ][ ] : [ ][ ]	
102	What is the name of the sick child?	NAME ________________________	

CLIENT AGE

NO.	QUESTIONS	CODING CLASSIFICATION	GO TO
103	What month and year was [NAME] born?	MONTH . . . . [ ][ ]<br>DON'T KNOW MONTH . . . . 98<br>YEAR . . . . [ ][ ][ ][ ]<br>DON'T KNOW YEAR 9998	
104	How old is [NAME] in completed months?	AGE IN MONTHS . . . . . . [ ][ ]<br>DON'T KNOW. 9 8	

SIGNS AND SYMPTOMS OF CURRENT ILLNESS

NO.	QUESTIONS	CODING CLASSIFICATION	GO TO
105	Has [NAME] had fever with this illness or any time in the past two days?	YES. . . . . 1<br>NO. . . . . 2<br>DON'T KNOW. 8	
106	Has [NAME] had a convulsion with this illness?	YES. . . . . 1<br>NO. . . . . 2<br>DON'T KNOW. 8	
107	Does [NAME] have cough or difficulty breathing with this illness?	YES. . . . . 1<br>NO. . . . . 2<br>DON'T KNOW. 8	
108	Can [NAME] drink, eat or breastfeed?	YES. . . . . 1<br>NO. . . . . 2<br>DON'T KNOW. 8	
109	Does [NAME] vomit everything when he/she eats or breastfeeds during this illness?	YES. . . . . 1<br>NO. . . . . 2<br>DON'T KNOW. 8	

110	Has [HE/SHE] had watery and frequent stools with this illness or any time in the past two days?	YES. . . . . 1<br>NO. . . . . 2<br>DON'T KNOW. 8	
111	Has [HE/SHE] been excessively sleepy during this illness?	YES. . . . . 1<br>NO. . . . . 2<br>DON'T KNOW. 8	
112	For what other reason(s) did you bring [NAME] to this health facility today?<br><br>CIRCLE ALL ITEMS THE RESPONDENT MENTIONS<br><br>PROBE: Anything else?	EAR PROBLEMS. . . . . A<br>SKIN SORE/PROBLEMS. . . . . B<br>INJURY. . . . . C<br>OTHER ______________ X<br>(SPECIFY)<br>NO OTHER REASON . . . . Y	
113	Has [NAME] been brought to this facility before for this same illness?<br><br>IF YES, ASK: How long ago was that?	WITHIN THE PAST WEEK. . . . . 1<br>WITHIN THE PAST 2-4 WEEKS. . . . 2<br>MORE THAN 4 WEEKS AGO. . . . . . 3<br>NO. . . . . 4<br>DON'T KNOW. 8	
114	How many days ago did the illness for which you brought [NAME] here begin?<br><br>IF LESS THAN 1 DAY, ENTER 00	DAYS AGO. . . . . [ ][ ]<br><br>DON'T KNOW. 98	

INFORMATION PROVIDED TO CARETAKER

115	Did the provider tell you what illness [NAME] has?	YES. . . . . 1<br>NO. . . . . 2<br>DON'T KNOW. 8	
116	What would you do if [NAME] does not get completely better or becomes worse?	RETURN TO FACILITY. . . . . 1<br>GO TO OTHER FACILITY. . . . . 2<br>GO TO OTHER HEALTH<br>WORKER OR /PHARMACY. . . . . . 3<br>GO TO TRADITIONAL HEALER. . . . 4<br>NOTHING, JUST WAIT. . . . . 5<br>DON'T KNOW. 8	
117	Did the provider tell you about any signs or symptoms you may see for which you must immediately bring the child back?<br><br>IF YES, ASK: Can you tell me what these are?<br><br>IF NECESSARY, PROBE:<br>Were there any serious symptoms or danger signs for which you were told to bring [NAME] back immediately?	FEVER . . . . A<br>BREATHING PROBLEMS . . . . . . B<br>BECOMES SICKER . . . . C<br>BLOOD IN STOOL . . . . D<br>VOMITING . . . . E<br>POOR/NOT EATING . . . . F<br>POOR/NOT DRINKING . . . . G<br>OTHER ______________ X<br>(SPECIFY)<br>NO, NONE . . . . Y<br>DON'T KNOW . . . . Z	
118	Did the provider tell you anything about bringing [NAME] back to the health facility for follow-up or non-emergency reasons?<br><br>IF YES:<br><br>Why were you to return?	MORE MEDICINES . . . . A<br>IF SYMPTOMS INCREASE OR<br>BECOME WORSE . . . . B<br>FOLLOW-UP APPOINTMENT. . . . . C<br>VIT. A SUPPLEMENTATION. . . . . . . D<br>LAB TEST RESULTS. . . . . E<br>CHILD ADMITTED. . . . . F<br>ROUTINE IMMUNISATION . . . . . . G<br>OTHER ______________ X<br>(SPECIFY)<br>NO. . . . . Y<br>DON'T KNOW . . . . Z	

TREATMENT AND CARETAKER COMFORT LEVEL

119	Did the provider give or prescribe any medicines for [NAME] to take at home?	YES, GAVE MEDS. . . . . 1<br>YES, GAVE PRESCRIPTION. . . . . . 2<br>GAVE MEDS AND PRESCRIPTION. . . . . 3<br>NO 4	4 → 124
120	ASK TO SEE ALL MEDICATIONS THAT THE CARETAKER RECEIVED AND ANY PRESCRIPTIONS THAT HAVE NOT YET BEEN FILLED.<br><br>CIRCLE THE RESPONSE DESCRIBING THE MEDICATIONS AND PRESCRIPTIONS YOU SEE.	HAS ALL MEDS. . . . . 1<br>HAS SOME MEDS, SOME UNFILLED PRESCRIPTIONS. . . . 2<br>NO MEDICATIONS SEEN, HAS PRESCRIPTIONS ONLY. 3	
121	Did a provider at the facility explain to you how to give these medicines to [NAME] at home?<br><br>IF “2” OR “8” SEND CLIENT BACK TO PROVIDER AT THE END OF THE INTERVIEW	YES. . . . . 1<br>NO. . . . . 2<br>DON’T KNOW. 8	
122	Do you feel comfortable or confident that you know how much of each medication to give [NAME] each day and for how many days to give it?<br><br>IF “2” OR “8” SEND CLIENT BACK TO PROVIDER AT THE END OF THE INTERVIEW	YES. . . . . 1<br>NO. . . . . 2<br>DON’T KNOW. 8	
123	Has [NAME] been given a dose of any of these medications here at the facility already?	YES. . . . . 1<br>NO. . . . . 2<br>DON’T KNOW. 8	
124	Did [NAME] receive an injection for treating the sickness here at the facility today?<br>IF NO, CHECK PRESCRIPTIONS AND RECORD IF THERE IS A PRESCRIPTION FOR AN INJECTION.	YES, RECEIVED INJECTION. . . . . . 1<br>YES, RECEIVED PRESCRIPTION FOR INJECTION. . . . . 2<br>NO . . . . 3<br>DON’T KNOW 8	
125	Did anyone at the health facility weigh [NAME] today?	YES . . . . 1<br>NO 2	
126	Did anyone talk to you today about [NAME]’s weight and how [NAME] is growing?	YES . . . . 1<br>NO 2	
127	Did any provider ask you today about the types of foods and amounts that you normally feed [NAME] when [NAME] is not sick?	YES . . . . 1<br>NO . . . . 2<br>CANNOT REMEMBER 8	
128	What did the provider tell you about feeding solid foods to [NAME] during this illness?	GIVE LESS THAN USUAL . . . . . . 1<br>GIVE SAME AS USUAL . . . . 2<br>GIVE MORE THAN USUAL . . . . 3<br>GIVE NOTHING/DON’T FEED . . 4<br>DIDN’T DISCUSS . . . . 6<br>NOT CERTAIN 8	
129	What did the provider tell you about giving fluids (or breast milk, if the child is breastfed) to [NAME] during this illness?	GIVE LESS THAN USUAL . . . . . . 1<br>GIVE SAME AS USUAL . . . . 2<br>GIVE MORE THAN USUAL . . . . 3<br>GIVE NOTHING/DON’T FEED . . 4<br>DIDN’T DISCUSS . . . . 6<br>DON’T KNOW 8	

130	Was [NAME] given a vaccination today?<br><br>IF YES, ASK TO SEE THE HEALTH CARD OR BOOKLET TO VERIFY.	YES, OBSERVED. . . . . 1<br>REPORTED, NOT SEEN. . . . . 2<br>NO. . . . . 3<br>DON'T KNOW. 8	

REFERRAL

131	Did the provider instruct you to take [NAME] to see another provider or to a laboratory in this facility for a finger or heel stick for blood to be taken for a test?	YES. . . . . 1<br>NO. 2			→ 134 (NO)
132	Did you take [NAME] to the provider or laboratory for the finger or heel stick?	YES. . . . . 1<br>NO. 2			→ 134 (NO)
133	Were you told the result of the test that was done?	YES. . . . . 1<br>NO. 2			
134	Did the provider instruct you to take [NAME] to see a provider in another facility, or for a laboratory test outside of this facility, for further care for [NAME]?	YES. . . . . 1<br>NO. 2			→ 136 (NO)
135	Regarding this referral, please tell me:	YES	NO	DK	
01	Were you given any paper or record to take with you for the referral?	1	2	8	
02	Were you told ***where*** to go for the referral?	2	2	8	
03	Were you told ***who*** to see for the referral?	1	2	8	
04	Were you told ***why*** you are to go for the referral?	1	2	8	
05	Do you intend to go to this (these) referral(s)?	1	2	8	
136	Did you take [NAME] to see another health provider or traditional healer before coming here?<br><br>IF YES, ASK: Whom did you see and where?<br><br>CIRCLE ALL THAT APPLY	YES, OTHER PROVIDER THIS FACILITY. . . . . A<br>YES, OTHER PROVIDER DIFFERENT FACILITY. . . B<br>YES, TRADITIONAL HEALER. . . . . . C<br>SAW NO ONE . . . . Y			

CLIENT RE-EXAMINATION

	**READ TO CLIENT:** As part of this interview, and in order to improve services that this and other facilities provide, I will like to take a few measurements on [CHILD]. It will only take a few minutes<br><br>As with the rest of the interview, whether you decide to let me take these measurements on [CHILD] is completely voluntary and will not affect services you receive during this or future visits. However, we are counting on your cooperation to obtain information to help improve service provision in general.<br><br>Do you have any questions at this time? Do I have your permission to proceed?<br><br>Interviewer's signature<br>(Indicates respondent's willingness to participate)	DAY __ __ MONTH __ __ YEAR 2 0 1 __	
150	May I begin the interview?	CLIENT AGREES . . . . 1<br>CLIENT REFUSES 2	2 → 201
151	**CHECK Q107 ABOVE**<br><br>DOES THE CHILD HAVE COUGH OR DIFFICULTY BREATHING WITH THIS CURRENT ILLNESS?	YES. . . . . 1<br>NO. 2	2 → 153
152	PERFORM A 60-SECOND RESPIRATORY RATE COUNT ON THE CHILD<br><br>ENSURE THAT THE CHILD IS CALM DURING THE 60-SECOND COUNT	RESPIRATORY RATE/MINUTE . . . . __ __ __	
153	EXAMINE THE CHILD FOR THE FOLLOWING SIGNS OF ANEMIA. CIRCLE ALL SIGNS THAT YOU SEE.	PALE PALM. . . . . A<br>PALE EYELIDS. B<br>PALE TONGUE. . . . . C<br>NONE OF THE ABOVE. . . . . Y	
154	MEASURE THE CHILD'S TEMPERATURE	TEMPERATURE IN °CELCIUS. . . . . . __ __ . __	
155	ASSESS THE CONSCIOUSNESS LEVEL OF CHILD. IS HE/SHE CONSCIOUS, LETHARGIC OR UNCONSCIOUS?<br><br>GENTLY AROUSE CHILD IF HE/SHE APPEARS TO BE SLEEPING<br><br>**NOTE: CONTACT A HEALTH CARE PROVIDER IF YOU FIND THE SICK CHILD TO BE EITHER LETHARGIC OR UNCONSCIOUS**	CONSCIOUS. . . . . 1<br>LETHARGIC/UNCONSCIOUS. 2	

2. Client Satisfaction

NO.	QUESTIONS	CODING CLASSIFICATION	GO TO
	Now I am going to ask you some questions about the services you received today. I would like to have your honest opinion about the things that we will talk about. This information will help improve services in general.		
201	How long did you wait between the time you arrived at this facility and the time you were able to see a provider for the consultation?	MINUTES . . . . [ ][ ][ ]<br>SAW PROVIDER IMMEDIATELY . . . . 000<br>DON'T KNOW 998	
202	Now I am going to ask about some common problems clients have at health facilities. As I mention each one, please tell me whether any of these were problems for you today, and if so, whether they were **major** or **minor** problems for you.		

NO.		MAJOR	MINOR	NO PROB-LEM	DK
01	Time you waited to see a provider	1	2	3	8
02	Ability to discuss problems or concerns about [CHILD'S] illness	1	2	3	8
03	Amount of explanation you received about the problem or treatment	1	2	3	8
04	Privacy from having others see the examination	1	2	3	8
05	Privacy from having others hear your consultation discussion	1	2	3	8
06	Availability of medicines at this facility	1	2	3	8
07	The hours of service at this facility, i.e., when they open and close	1	2	3	8
08	The number of days services are available to you	1	2	3	8
09	The cleanliness of the facility	1	2	3	8
10	How the staff treated you	1	2	3	8
11	Cost for services or treatments	1	2	3	8

NO.	QUESTIONS	CODING CLASSIFICATION	GO TO
203	Are you a part of any prepayment plan (such as medical aid, insurance or a similar program) or institutional arrangement that pays for some or all of the services you receive at this or any other facility?	YES. . . . . 1<br>NO. . . . . 2<br>DON'T KNOW. 8	
204	Were you charged, or did you pay fees for any services your received or were provided today?	YES . . . . 1<br>NO 2	 → 206

205	What is the total amount you paid for all services or treatments you received at this facility today?	TOTAL AMOUNT [ ][ ][ ][ ][ ][ ]<br><br>DON'T KNOW 999998	
206	Is this the closest health facility to your home?	YES. . . . . 1<br>NO. . . . . 2<br>DON'T KNOW. 8	→ 208<br><br>→ 208
207	What was the main reason you did not go to the facility nearest to your home?<br><br>IF CLIENT MENTIONS SEVERAL REASONS, PROBE FOR THE MOST IMPORTANT, OR MAIN REASON.	INCONVENIENT OPERATING<br>HOURS . . . . 01<br>BAD REPUTATION . . . . 02<br>DON'T LIKE PERSONNEL . . 03<br>NO MEDICINE . . . . .04<br>PREFERS TO REMAIN<br>ANONYMOUS . . . . 05<br>IT IS MORE EXPENSIVE . . . . 06<br>WAS REFERRED . . . . .07<br>OTHER. . . . . 96<br>DON'T KNOW 98	
208	In general, which of the following statements best describes your opinion of the services you either received or were provided at this facility today<br><br>READ ALL STATEMENTS, CIRCLE ONLY ONE<br><br>01) I AM **VERY SATISFIED** WITH THE SERVICES I RECEIVED IN FACILITY . . . . . . 1<br><br>02) I AM **MORE OR LESS SATISFIED** WITH THE SERVICES I RECEIVED. . . . . 2<br><br>03) I AM **NOT SATISFIED** WITH THE SERVICED I RECEIVED 3		
210	Will you recommend this health facility to a friend or family member?	YES. . . . . 1<br>NO. . . . . 2<br>DON'T KNOW. 8	

3. Client Personal Characteristics

NO.	QUESTIONS	CODING CLASSIFICATION	GO TO
Now I am going to ask you some questions about yourself. I would like to have your honest responses as this information will help to improve services in general.			
301	What is your relationship to [SICK CHILD]?	MOTHER . . . . 1<br>FATHER . . . . 2<br>SIBLING . . . . 3<br>AUNT OR UNCLE . . . . 4<br>GRAND MOM/GRAND DAD. . . . . 5<br>OTHER ____________ 6<br>(SPECIFY)	
302	How old were you at your last birthday?	AGE IN YEARS . . . . [ ][ ]<br>DON'T KNOW. 98	
303	Have you ever attended school?	YES . . . . 1<br>NO 2	 2 → 305
304	What is the highest level of school you attended?	PRIMARY. . . . . 1<br>POST-PRIMARY/VOCATIONAL. 2<br>SECONDARY/A-LEVEL. . . . . . . 3<br>COLLEGE (MIDDLE LEVEL). . . 4<br>UNIVERSITY. 5	2–5 → 306
305	Do you know how to read or how to write?	YES, READ AND WRITE . . 1<br>YES, READ ONLY . . . . . 2<br>NO 3	
306	RECORD THE TIME THE INTERVIEW ENDED	[ ][ ] : [ ][ ]	
	Thank you very much for taking the time to answer my questions. Once again, any information you have given will be kept completely confidential. Have a good day!		

Interviewer's comments:
